# Electromagnetic and optical responses of a composite material comprising individual single-walled carbon-nanotubes with a polymer coating

**DOI:** 10.1038/s41598-020-66247-8

**Published:** 2020-06-09

**Authors:** Mikhail V. Shuba, Dzmitry Yuko, Polina P. Kuzhir, Sergey A. Maksimenko, Vitaly K. Ksenevich, Sung-Hwan Lim, Tae-Hwan Kim, Sung-Min Choi

**Affiliations:** 1 Institute for Nuclear Problems, Belarusian State University, 220006 Minsk, Belarus; 2 Tomsk State University, 634050 Tomsk, Russia; 3 Institute of Photonics, University of Eastern Finland, FI-80101 Joensuu, Finland; 4 Belarusian State University, Physics Faculty, 220030 Minsk, Belarus; 5 Korea Advanced Institute of Science and Technology, Department of Nuclear and Quantum Engineering, Daejeon, 34141 Republic of Korea; 6 Jeonbuk National University, Department of Qauntum System Engineering, 567 Baekje-daero Deokjin-gu Jeonju-si, Jeollabuk-do, 54896 Republic of Korea

**Keywords:** Carbon nanotubes and fullerenes, Carbon nanotubes and fullerenes

## Abstract

The composites and thin films comprising individual single-walled carbon nanotubes with a polymer coating (p-CNTs) have been prepared and their electromagnetic responses have been studied in a wide range from low-frequency (25–10^7^ Hz) up to the infrared region. In spite of the high volume fraction of the nanotubes (up to 3.3%), the polymer coating prevents direct p-CNT contacts and the formation of the percolation network in those composites, so that p-CNTs interact only via the electromagnetic coupling. Thereby it is an ideal model system to verify experimentally the fundamental issues related to carbon nanotube electromagnetics, such as the influence of inter-tube electron tunneling on the localized plasmon resonance in the terahertz range, or the infrared absorption enhancement of polymer molecules attached to the nanotube surface. Along with addressing the fundamentals, applied carbon nanotube electromagnetics got insights important for the applications of p-CNT based composites as dielectric media in the terahertz regime. In particular, we found that the real part of the permittivity of the p-CNT film in the terahertz range is rather competitive, i.e. 8–13, however the loss tangent is not so small (0.4–0.6) as has been predicted. The way to increase p-CNT terahertz performance is also discussed.

## Introduction

Among carbon nanoparticles, metallic single-walled carbon nanotubes (CNTs) have a huge aspect ratio (10^2^–10^7^) and high conductivity that both provide their strong interaction with the electromagnetic and optical radiations^1–4^. The high kinetic inductance of a single-walled CNT causes the slowed-down surface wave propagation along the tube in the range of the intraband electron transitions (<40 THz)^5,6^. The excitation of the standing surface waves by a plane wave manifests itself as antenna resonances in the polarizability spectrum of an individual finite-length CNT^3^. The first antenna resonance also called as localized plasmon resonance (LPR) appears as a broad terahertz peak in the conductivity spectra of the CNT films^7–9^.

The scattering theory has been developed for individual and bundled CNTs^3,10^, curved CNT^11^, CNT with a mesoscopic insertion^12^ and dielectric coating^13^. The electromagnetic response of a CNT composite has been theoretically described from radio-frequency up to the visible range with the Waterman-Truell approach^8^ that is valid for non-interacting inclusions, i.e. when the electromagnetic interaction and electron tunneling coupling between the tubes are small. However, theoretical study of the mutual impedance between two carbon nanotubes^14,15^ in radiofrequency and microwave ranges shows that the electromagnetic interaction between CNTs is essential if the distance between them is less than 0.02*λ*^15^ (*λ* is a wavelength of the electromagnetic wave in the surrounding medium). Moreover, the CNTs tend to aggregate and the percolation in the composite media occurs even at a low CNT volume fraction (<0.5%)^16,17^. Consequently, the intertube electron tunneling occurs in realistic composites.

Here, we propose for the first time a unique composite material comprising individual single-walled CNTs; each CNT is covered with a polymer coating preventing the percolation effect in the composite material even at 3.3% CNT volume fraction. Hereinafter, we shall refer to CNTs with the polymer coating as p-CNTs. These tubes can interact with each other only by means of the electromagnetic field. In this paper, we present the study of the electromagnetic and optical properties of p-CNT composites and films. This material is an excellent tool to check two following theoretical predictions:

(i) Due to the high value of the real part and low value of the imaginary part of the polarisability of short-length CNTs, they can be considered as building blocks for high dielectric constant material in the terahertz range^18^. The calculation based on the Waterman-Truell approach predicts that the composite media comprising individual isolated short-length CNTs may have a high real part of the permittivity and low loss tangent in the terahertz range.
(ii) The scattering theory predicts the near-field enhancement effect in individual CNTs^13^. This effect can lead to the absorption enhancement of the molecules adsorbed on the surface of CNTs^4^. Note that significant absorption enhancement in the middle-infrared (mid-IR) range has been already reported in^19^. The absorption enhancement in the microwave range has been also observed in the CNT suspension^20^.

Both these predictions will be checked using p-CNTs. Also, we shall show how the intertube electron tunneling modifies the terahertz conductivity peak of the CNT films.

## Results and discussions

**Figure 1:**
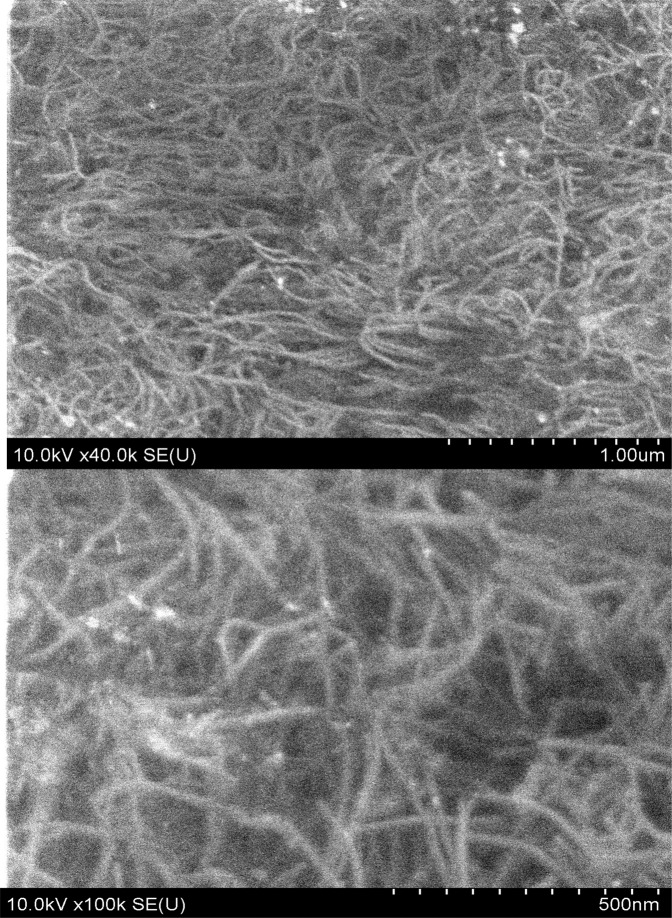
SEM images of the p-CNT composite with 5 wt% CNTs at different magnifications.

**Figure 2:**
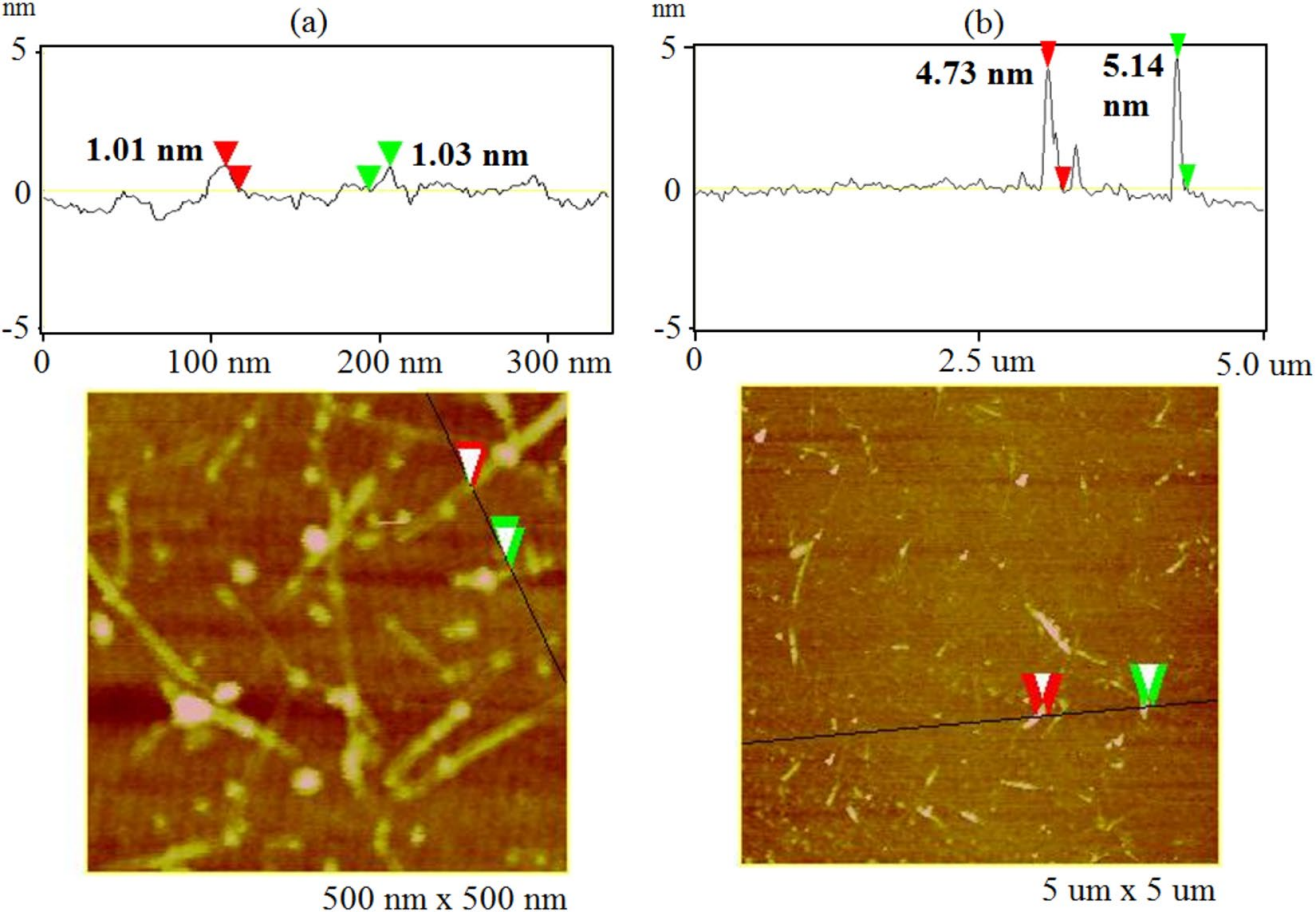
AFM images and sectional analysis of (**a**) bare CNTs and (**b**) p-CNTs.

Two types of p-CNT materials with 1.5 and 5 wt% single-walled CNTs were fabricated; then they were used to produce composites and films comprising p-CNTs (see Methods). A typical scanning electron microscopy (SEM) image of the p-CNT composite with 5 wt% CNTs is represented in Fig. 1 (the SEM images of 1.5 wt% CNT composite can be found as Supplementary Fig. S1). One can see that p-CNTs are in contact with each other and their lengths do not exceed 1 *μ*m. The space between p-CNTs is occupied by air. Here we would like to stress that the tubes are mostly individual, as the bundles were removed by strong centrifugation at ca. 111000 g for 4 h (see Methods). This is confirmed by the atomic-force microscopy (AFM). Typical AFM images for bare CNTs and p-CNTs are shown in Fig. 2(a,b), respectively.

As obtained from AFM measurements, the diameter of bare CNTs is 1–1.4 nm, whereas the total diameter of p-CNTs is 4.5–5.5 nm^21,22^. This means that the thickness of the coating varies in the range 1.7–2.5 nm. The density of a polymer is about 1 g/cm^−3^ ^23^. After simple calculations made for CNT with a diameter of 1 nm and the thickness of the coating being 2 nm, we found that the ratio of the CNT mass to the mass of the polymer coating equals 0.11. Then, for the composite with 5 wt% CNTs, about 50% of the polymer is attached to the CNT surface, and about 50% of the polymer is free and does not cover the CNTs.

Let us also stress that fabricated p-CNT films comprise densely packed CNTs. The CNT volume fraction in 5 wt% p-CNT film has been estimated as *F* = 3.3%. The rest 96.7% of the volume is occupied by polymer. For the hypothetical ordered array of oriented CNTs, the value *F* = 3.3% corresponds to the average distance of 5.5 nm between axes of neighboring CNTs. Such a small distance may lead to a strong electromagnetic interaction between the neighboring tubes.

**Figure 3:**
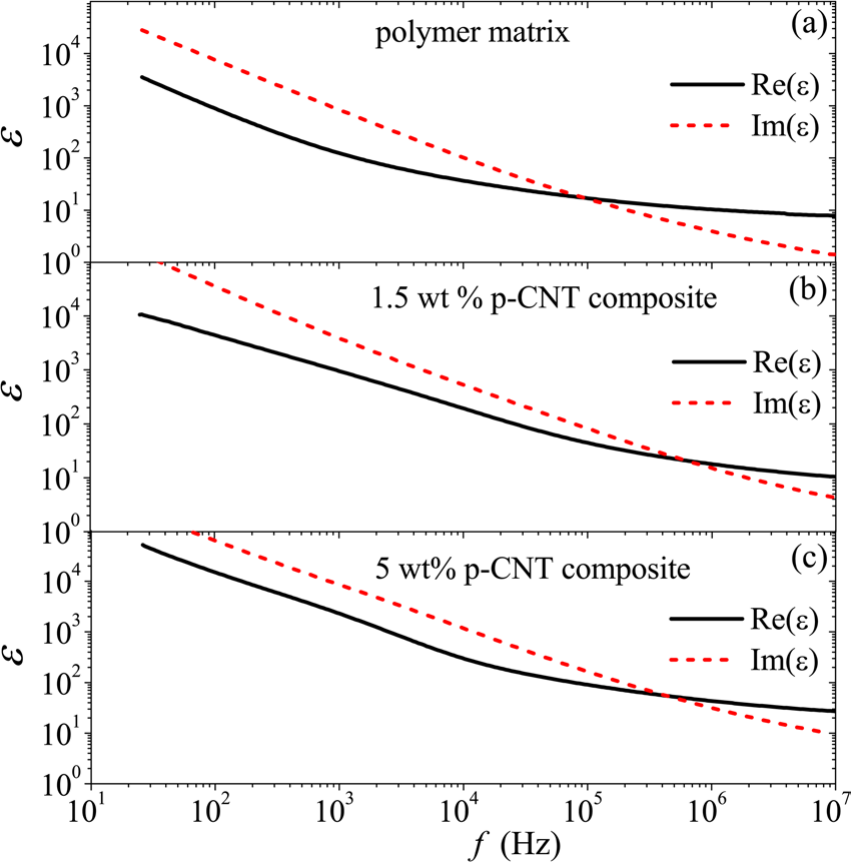
Frequency dependence of the real and imaginary parts of the permittivity for (**a**) polymer matrix, (**b**) 1.5 wt%, and (**c**) 5 wt% p-CNT composites.

**Figure 4:**
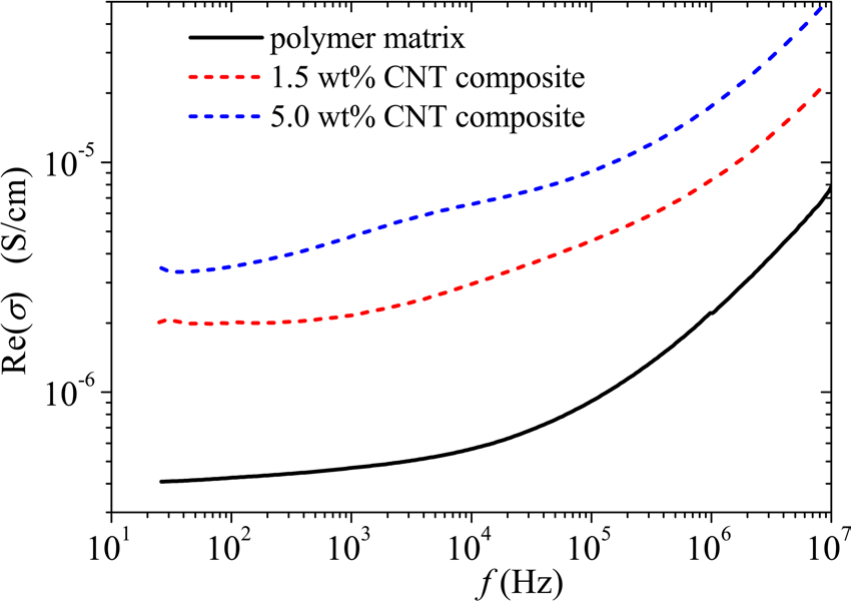
Frequency dependence of the real part of the conductivity for (**a**) polymer matrix, (**b**) 1.5 wt%, and (**c**) 5 wt% p-CNT composites.

Figures 3 and 4 show the permittivity and conductivity spectra for the polymer matrix, 1.5 wt% and 5 wt% p-CNT composites in the range 25 Hz–10 MHz. All the samples demonstrate strong frequency dependence of the permittivity. Since Re(*ε*) < Im(ε) below 10^5^ Hz, all the samples are conductive in that range. In accordance with the Kramers-Kronig relation, the high imaginary part of the permittivity is accompanied by the high real part of the permittivity (see Fig. 3). Above 1 MHz, the samples can be considered as a dielectric with rather high losses, as Re(*ε*)  >Im(ε) is true for them. Rather strong frequency dependence of the conductivity of p-CNT composites in Fig. 4 means the absence of the percolation effect. One may conclude that the polymer coating prevents the electron tunneling between the tubes. Further, we shall consider the high-frequency dielectric properties of 5 wt% p-CNT film having the highest (3.3%) volume fraction of CNTs (see details of the film fabrication and measurement techniques in Methods).

**Figure 5:**
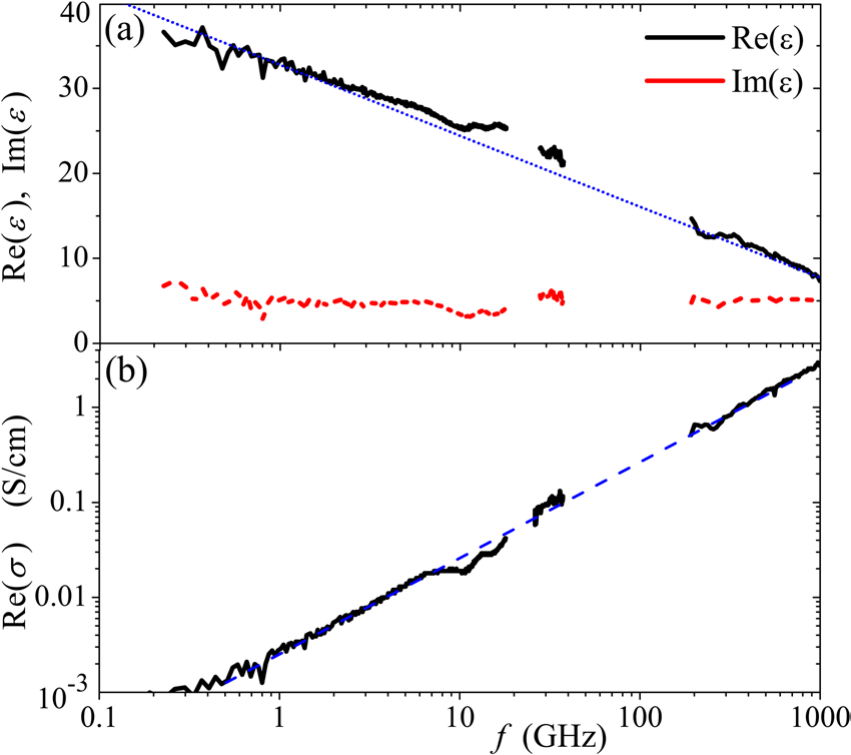
Frequency dependence of (**a**) Re(*ε*), Im(ε), and (**b**) Re(*σ*) for CNT polymer films. Dotted line in (**a**) and dashed line in (**b**) demonstrates fitting of the experimental data with a function Re(*ε*) = 108 − 3.63 ln(*f*), where *f* is in Hz, and Re(*σ*) ∝ *f*, respectively.

Figure 5 demonstrates the frequency dependence of the permittivity and conductivity for 5 wt% p-CNT film. One can see, that the value of Im(ε) remains almost constant and equal to 5 in a wide range 0.2–1000 GHz, whereas the value of Re(*ε*) has approximately logarithmic frequency dependence; dotted line in Fig. 5(a) demonstrates fitting of the experimental data with a function Re(*ε*) = 108 − 3.63 ln(*f*), where *f* is in Hz. The conductivity of the composite is directly proportional to the frequency, see data fitted with a dashed line in Fig. 5(b). Dielectric loss tangent, tan*δ* = Im(*ε*)/Re(*ε*), is equal to 0.14 at 0.2 GHz and it increases monotonically with frequency and reaches the value of 0.63 at 1 THz. Let us notice that the permittivity of the polymer matrix in the range 0.2–1 THz is about 2.1 + 0.15*i*.

As predicted in^18^, the composite comprising individual short-length CNTs non-interacting with each other is expected to have a high real part of the permittivity and low loss tangent in the terahertz range. For example, it is expected that Re(*ε*) = 1000 and tan*δ* = 0.05 at frequency 0.5 THz for the composite with CNT volume fraction *F* = 20% and tube length *L* = 0.4 *μ*m^18^. Moreover, the theory predicts an increase of the value of Im(*ε*) with frequency. However, for realistic composite, as shown in Fig. 5, it does not occur. For p-CNT film, we obtain Re(*ε*) ≈ 10 and tan*δ* = 0.47 at *f* = 0.5 THz, *F* = 3.3%, and *L* < 1 *μ*m. The reason for the discrepancy between the theory and experiment could be that the electromagnetic interaction between neighboring CNTs has not been taken into account in the calculations, though it may lead to additional energy dissipation and to the modification of the local field acting on the CNTs. Let us notice that the value of Re(*ε*) can be increased by 2–3 times by orienting p-CNTs in the film.

**Figure 6:**
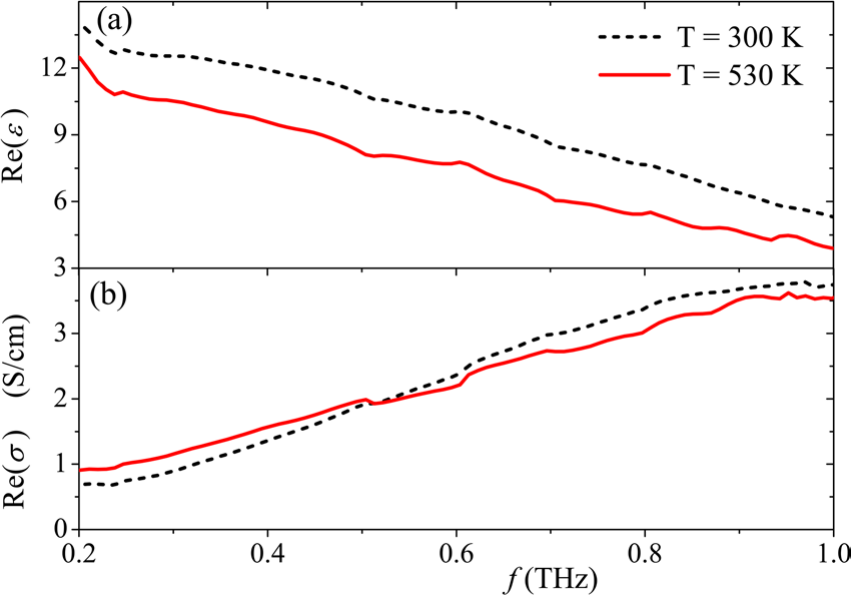
Frequency dependence of the real part of (**a**) the permittivity and (**b**) conductivity of the p-CNT film at 300 and 530 K.

Figure 6 shows the frequency dependence of the real part of the permittivity and conductivity of the p-CNT film in the range 0.2–1 THz at different temperatures, 300 and 530 K. One can see that heating leads to a decrease of the value Re(*ε*). The sign of the value ∂Re(*σ*)/∂*T* changes at 0.5 THz. As discussed in^17^, the heating leads to an increase of the electron relaxation rate resulting in a broadening of the terahertz conductivity peak. The peak frequency increases as tube length decreases. Depending on the length of the nanotubes, the spectral position of the LPR peak with respect to the range 0.2–1 THz can be different. Therefore, it is expected that ∂Re(*σ*)/∂*T* > 0 for short-length tubes (<0.3 *μ*m), and ∂Re(*σ*)/∂*T* < 0 for long-length tubes (>5 *μ*m) in considered frequency range (see Fig. 1 in^17^). p-CNTs have lengths of about 0.5 *μ*m resulting in a sign variation of the value ∂Re(*σ*)/∂*T*, as shown in Fig. 6(b).

In order to show the influence of the intertube electron tunneling on the conductivity spectra of a CNT composite material, we prepared two 1.5 wt% p-CNT films of the same thickness (see fabrication of the CNT-1 and CNT-2 films in Methods). In the first one (CNT-1), all the tubes are almost isolated from one another, and its dc-conductivity is 4 × 10^−5^ S/cm. In the second film (CNT-2), the polymer coating of CNTs is partly damaged and CNTs have electrical contacts with each other so that the dc-conductivity of the film is 4 × 10^−3^ S/cm; it is 200 times higher than that for CNT-1 film.

**Figure 7:**
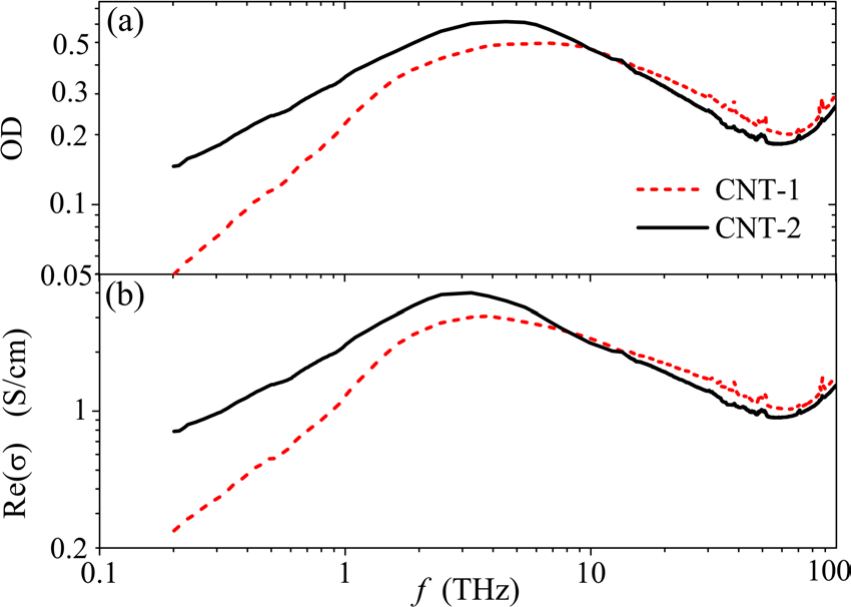
Frequency dependence of (**a**) the optical density (OD) and (**b**) real part of the conductivity for 1.5 wt% p-CNT films of low (CNT-1) and high (CNT-2) dc-conductivity.

Figure 7 shows the frequency dependence of the optical density and real part of the conductivity for the CNT-1 and CNT-2 films in the terahertz and far-IR ranges. All the spectra have a broad terahertz peak due to the LPR phenomena in CNTs. The conductivity on the low frequency side of the peak is higher for CNT-2 than for CNT-1. One may conclude that this part of the spectra is sensitive to the electron tunneling between the tubes. Moreover, one can see from Fig. 7 that an increase of a number of the electrical contacts between nanotubes leads to weaker frequency dependence of the conductivity on the low-frequency side of the LPR. In particular, in the range 0.2–2 THz, the dependence Re(*σ*(*f*)) follows power law with the exponents 1.0 and 0.65 for CNT-1 and CNT-2, respectively. Similar tendencies have been illustrated for hybrid films comprising single-walled CNTs and WS_2_ nanotubes at different fractions of CNTs^17^. The terahertz conductivity peak is slightly modified at the central frequency and on the high-frequency side. This means that the intertube electron tunneling influences weakly the LPR in the tubes. Thus, we may conclude that the Waterman-Truell homogenization approach, that neglects intertube interaction, can be applied to describe the conductivity spectrum of CNT-based composite at and above the LPR frequency.

Since the p-CNTs are distributed homogeneously in the film, and about 50% of the polymer in 5 wt% p-CNT film is non-covalently attached to the tube surfaces, they can be considered as a great tool to check the effect of IR absorption enhancement of polymer molecules on CNT surfaces. This effect originates from the enhancement of the field near the surface of CNTs. The standing surface waves are excited in CNTs exposed to the plane incident wave; this causes the near-field enhancement effect^4^.

**Figure 8:**
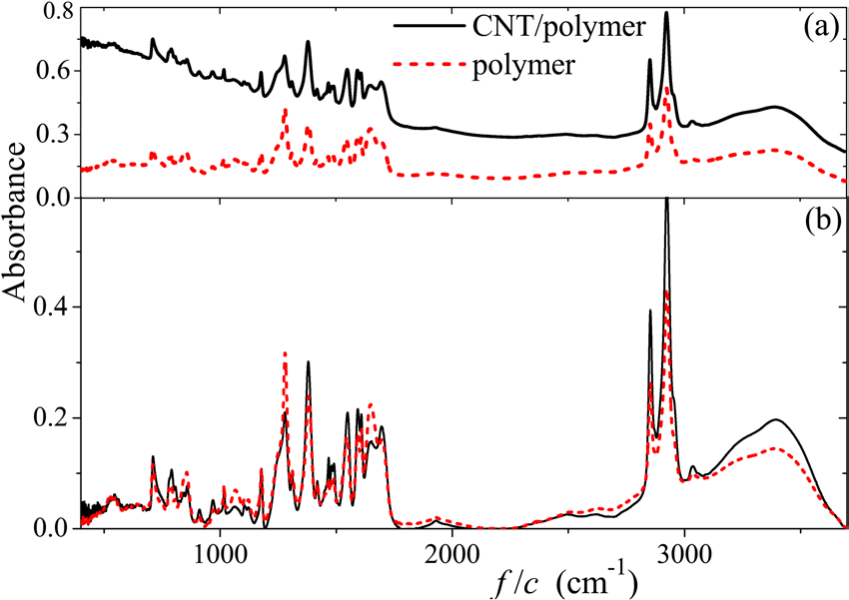
Mid-IR absorbance spectra of the polymer and p-CNT films (**a**) before and (**b**) after the subtraction of a background.

The experimental observation of several times absorption enhancement for thymine-CNT complexes on a gold film has been reported in^19^. In order to check if this effect occurs in our samples, we have obtained the mid-IR absorbance spectra for the polymer and p-CNT films of the same thickness (see Fig. 8). As shown in Fig. 8(a), there are many absorption bands associated with different stretching vibrations. Simultaneously, there is also a background which is rather large for p-CNT film, it is associated with the absorption in CNTs. Figure 8(b) shows the absorption bands after background subtraction for both samples. One can see from Fig. 8(b) that there is no strong difference in the absorbance spectra of the polymer molecules with and without CNTs. Therefore, we cannot report on the absorption enhancement effect in the region above 18 THz (600 cm^−1^). As shown in^4^, the near-field enhancement effect is strong at and below the frequency of the LPR. However, it becomes weaker at higher frequencies (see Fig. 4 in^4^). For our samples the LPR frequency is 3 THz (see Fig. 7). This explains why we could not observe the absorption enhancement effect in the range 18–52 THz.

In conclusion, dielectric and electromagnetic properties of the composite materials and films comprising a high volume fraction of individual single-walled CNTs with polymer coating have been measured and analyzed in a wide frequency range (from 25 Hz up to 100 THz). Polymer coating prevents aggregation of the tubes, and the percolation effect does not occur even at a high volume fraction of the inclusions (3.3%). p-CNT material demonstrates dielectric behavior in the microwave and terahertz ranges. The real part of the permittivity of the p-CNT film in the terahertz range is not so high and the loss tangent is not so small as predicted recently in^18^. The reason could be the electromagnetic interaction between the CNTs that has not been taken into account in the theoretical model.

Comparison between the p-CNT films with different numbers of intertube contacts demonstrates that the intertube tunneling strongly influences the low-frequency side of the terahertz conductivity peak and slightly modifies its central part and high-frequency side. This means that the interaction between the CNTs in the composite can be omitted and the simple Waterman-Truell approach can be applied for the calculation of the effective conductivity of the CNT-based composites at and above the LPR frequency.

Though about 50% of the polymer molecules are attached to the CNT surface in 5 wt% p-CNT film, we have not observed the effect of the absorption enhancement for those molecules in the mid-IR range.

## Methods

### Materials

HiPco (high-pressure CO conversion) single-walled carbon nanotubes (CNTs, purity >95 wt%, NanoIntegris), Dowex Monosphere 550 A (OH) anion exchange resin (Sigma-Aldrich), cetyltrimethylammonium bromide (CTAB, Sigma-Aldrich), 4-vinylbenzoic acid (VBA, Sigma-Aldrich), water soluble free-radical initiator 2,2′-azobis (2-(2-imidazolin-2-yl) propane) dihydrochloride (VA-044, Wako Chemicals), D_2_ O (99.9 mol% deuterium enriched, Cambridge Isotope Laboratory) were used as received from the companies. H_2_O was purified using a Millipore Direct Q system (electrical resistivity 18.2 MΩ) prior to use. Cetyl-trimethylammonium hydroxide (CTAOH) was synthesized by replacing Br^−^ of CTAB with OH^−^ using the anion exchange resin. Cetyl-trimethylammonium 4-vinylbenzoate (CTVB) was synthesized through neutralization of VBA with a same stoichiometric amount of CTAOH followed by repeated crystallization^21,22^.

*The non-covalently functionalized CNTs* (p-CNTs) were prepared as described elsewhere^21,22^. Briefly, HiPco single-walled CNTs (2 mg/mL) and the CTVB (5 mg/mL) were mixed in D_2_O and sonicated for 1 hour at 60 °C to exfoliate the SWNT bundles, which results in individually isolated CNTs with an adsorbed monolayer of CTVB. The counterions (VB^−^) of CTVB were then polymerized using the free radical initiator (VA-044) at a polymerization temperature of 60 °C, permanently fixing the CTVB monolayer to the CNTs. To separate individually functionalized CNTs from the bundled ones, the suspension was ultracentrifuged at ca. 111000 g for 4 h.

The upper 70% of the suspension was then decanted and followed by freeze-drying at −55 °C for 3 days. The obtained p-CNT material has many pores of about 10–100 *μ*m in size and has a density of about 0.2 g/cm^3^. Characterization of p-CNTs with UV-vis-NIR spectroscopy, atomic-force microscopy, and small-angle neutron scattering measurements is presented in previous reports^21,22^. Two p-CNT materials with 5 and 1.5 wt% CNTs have been produced.

### p-CNT composite fabrication

The plate was cut off from the p-CNT material and then it was compressed up to the thickness of 0.5–1 mm and density ≈0.9 g/cm^3^ in order to remove all the pores. We shall refer to the compressed plate as a p-CNT composite.

### p-CNT film fabrication

p-CNT material was dispersed by ultrasonic treatment (Ultrasonic device UZDN-2T, 44 kHz, maximum power) for 10 min in water. The suspension of p-CNTs was centrifuged at 8000 g for 20 min and then it was filtrated through a cellulose nitrate membrane filter (0.2 *μ*m pore size, the thickness of 100 *μ*m) causing a p-CNT film to form on the filter. The maximal film thickness that can be reached is 10 *μ*m, as thick polymer film does not transmit water and it stops the filtration process. The film thickness was calculated knowing the mass and density of the material and the surface area of the film. Both dc and ac conductivity of the p-CNT film was found to be higher than that for a p-CNT composite. This is probably due to the preferred orientation of the tubes parallel to the film surface and higher densification of the material.

In order to bind the tubes withing the film and avoid dissolving of the film with acetone, water or alcohol, the filter with the p-CNT film was heated at 100 °C for 30 min. The filter was then dissolved with acetone and the p-CNT film was transferred on the metallic frame to obtain a free-standing film for infrared (IR) and terahertz measurements. Microwave measurements have been done for the p-CNT film on the filter, as the latter is transparent in the microwave range.

### Fabrication of the CNT-1 and CNT-2 films

The polymer coating of p-CNTs was partly damaged by ultrasonic treatment of the p-CNT aqueous suspension at 90 °C for 5–10 min. We fabricated two 1.5 wt% p-CNT films, CNT-1 and CNT-2, comprising non-damaged and damaged p-CNTs, respectively. The intertube electrical contacts occur between damaged p-CNTs causing 200 times higher dc conductivity for CNT-2 than for CNT-1 film. SEM images of the CNT-1 and CNT-2 film cannot demonstrate their morphology, as CNTs are hidden by free polymer (see Supplementary Figs. S1–S2). Raman spectra of CNT-1 and CNT-2 films are found to be identical; they indicate high crystalline quality of CNTs in both films (see Supplementary Fig. S3).

*The AFM measurements* were carried out using a VEECO AFM instrument (Nanoman, SECPM). The sample for the AFM measurements was prepared by spin-coating of a p-CNT suspension at 4000 rpm for 1.5 min onto silicon wafers. The sample of bare CNTs was obtained by burning the p-CNTs on silicon wafers at 330 °C for 4 h to remove the CTVB coating from CNTs.

### Measurement techniques

The complex transmission of the films at normal incidence was measured in the frequency range *f* ∈ [0.2, 1] THz using time-domain terahertz spectrometer (THz-TDS, EKSPLA, Vilnius Lithuania). The permittivity *ε* and conductivity Re(*σ*) = 2*πfε*_0_Im(*ε*) of the films was then calculated using the Fresnel equations for a dielectric slab^24^; *ε*_0_ = 8.85 × 10^−12^ Fm^−1^. To investigate the temperature dependence of the terahertz conductivity of p-CNT film in the range 300–530 K in air, we employed the THz-TDS with a home-made furnace-style heating sample holder. Transmittance spectra of the films were obtained using a THz-TDS (0.2–2.0 THz, EKSPLA), and a Fourier-transform IR spectrometer Vertex 70 (2–100 THz, Bruker). The conductivity spectra of the films were calculated from the transmittance spectra using the Kramer-Kronig relation^25^. The microwave conductivity measurements were carried out in the ranges 0.1–18 GHz and 26–36 GHz by waveguide method^26^ using a vector analyzer R4M-18 (MICRAN, Tomsk, Russia) and a scalar network analyzer R2–408R (ELMIKA, Vilnius, Lithuania), respectively. Dielectric properties of the composites in the range 25 Hz–10 MHz was measured with a broadband analyzer 7–28 (MNIPI, Belarus). The electrical dc conductivity of p-CNT films was measured using a four-point linear probe technique.

## Supplementary information

Supplementary Information.
