# Supplementary material for: Electromagnetic and optical responses of a composite material comprising individual single-walled carbon-nanotubes with a polymer coating
Source: Sci Rep. 2020 Jun 9;10:9361. doi: 10.1038/s41598-020-66247-8 (PMC7283472; doi:10.1038/s41598-020-66247-8)
Supplement: Supplementary file 1 — Supplementary Information. [file 41598_2020_66247_MOESM1_ESM.pdf]

Electromagnetic and optical responses of a composite material comprising individual single-walled carbon-nanotubes with a polymer coating

Mikhail V. Shuba[1,2]*, Dzmitry Yuko[1], Polina P. Kuzhir[3,1], Sergey A. Maksimenko[1,2], Vitaly K. Ksenevich[4], Sung-Hwang Lim[5], Tae-Hwan Kim[5,6], and Sung-Min Choi[5]

[1]Institute for Nuclear Problems, Belarusian State University, 220006, Minsk, Belarus
[2]Tomsk State University, 634050, Tomsk, Russia
[3]Institute of Photonics, University of Eastern Finland, FI-80101 Joensuu, Finland
[4]Belarusian State University, Physics Faculty, 220030, Minsk, Belarus
[5]Korea Advanced Institute of Science and Technology, Department of Nuclear and Quantum Engineering, Daejeon, 34141, Republic of Korea
[6]Jeonbuk National University, Department of Qauntum System Engineering, 567 Baekje-daero Deokjin-gu Jeonju-si, Jeollabuk-do, 54896, Republic of Korea

* Correspondence to mikhail.shuba@gmail.com

Supplementary Information

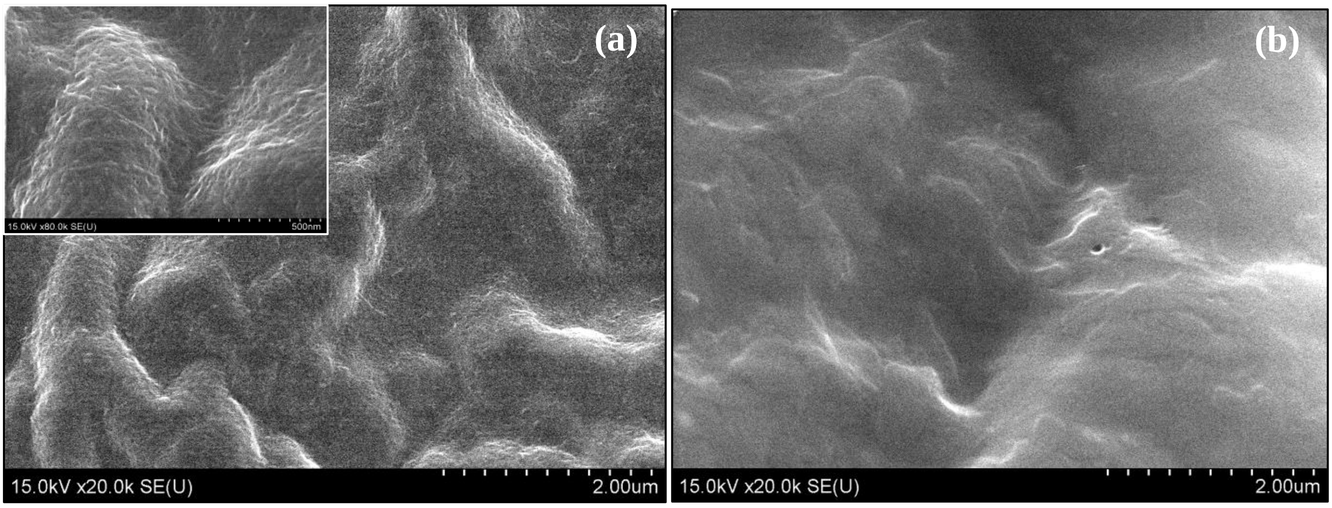

Figure S1. SEM images of (a) p-CNT composite at 1.5 wt% CNTs and (b) pure polymer matrix. Due to the presence of CNTs, the surface of CNT composite is not as smooth as the surface of pure polymer matrix. We estimated that, for 1.5 wt% p-CNT composite, 15% of the polymer is attached to CNTs, whereas 85% of the polymer is free.

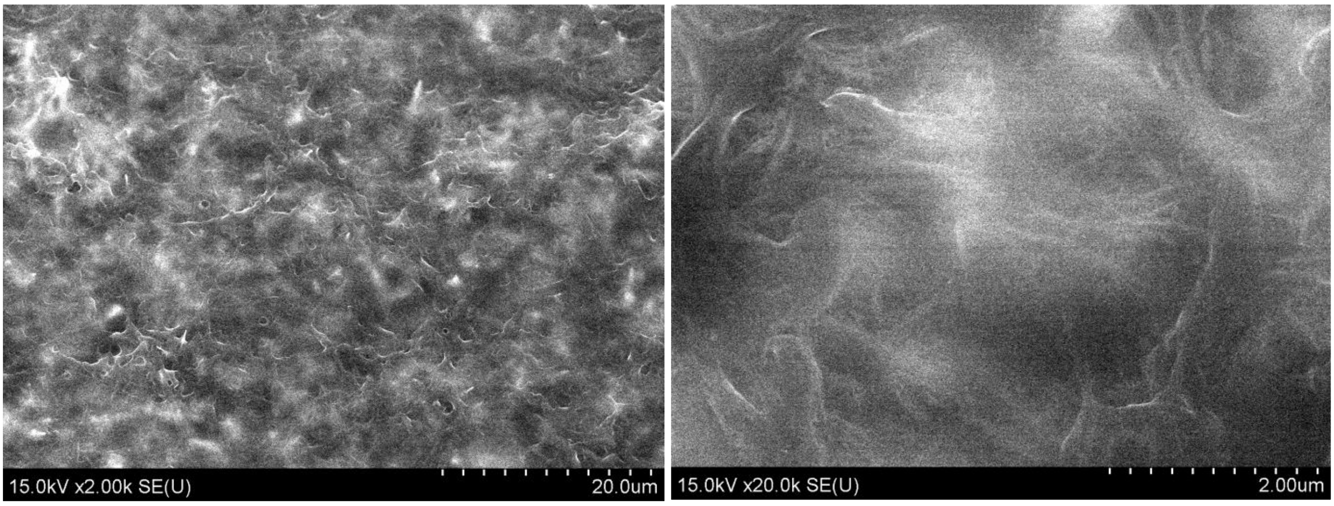

Figure S2. SEM images of the CNT-1 film at different magnifications. Similar images have been obtained for CNT-2 film. Since CNT-1 and CNT-2 films contain only 1.5 wt% of CNTs, carbon nanotubes are hidden under the polymer. Therefore, SEM images cannot represent the tube distribution inside the films.

We found that Raman spectra of the CNT-1 and CNT-2 films are the same. As an example, Raman spectrum for the CNT-1 film is shown in Fig. S3. The spectrum contains G-band (1550–1600 cm^{-1}) originating from tangential vibrations of sp^2-hybridized carbon atoms and D-band (1310–1325 cm^{-1}) which is associated with disorder-induced symmetry-lowering effects. The ratio of the D-band intensity to the G-band intensity is about 0.09 indicating small amount of sidewall defects in CNTs.

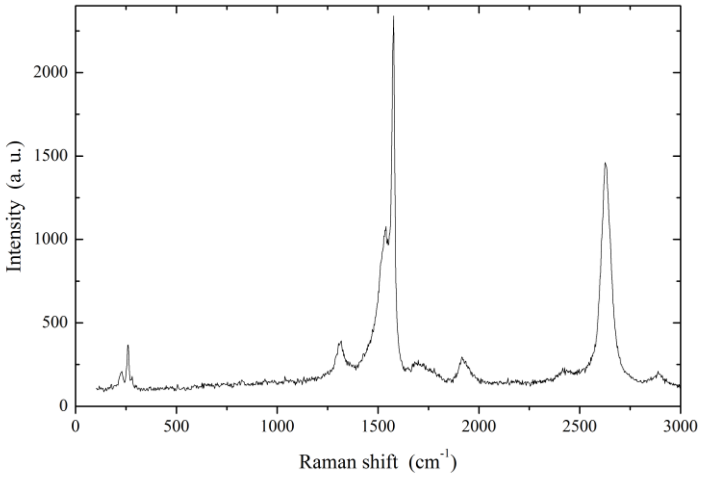

Figure S3. Raman spectrum of the CNT-1 film.
